# Systems-Based Training in Graduate Medical Education for Service Learning in the State Legislature in the United States: Pilot Study

**DOI:** 10.2196/mededu.7730

**Published:** 2017-10-17

**Authors:** Shikhar H Shah, Maureen D Clark, Kimberly Hu, Jalene A Shoener, Joshua Fogel, William C Kling, James Ronayne

**Affiliations:** ^1^ College of Medicine University of Illinois at Chicago Chicago, IL United States; ^2^ Library of the Health Sciences University Library University of Illinois at Chicago Chicago, IL United States; ^3^ Department of Medical Education College of Medicine University of Illinois at Chicago Chicago, IL United States; ^4^ Department of Internal Medicine College of Medicine University of Illinois at Chicago Chicago, IL United States; ^5^ Department of Pediatrics College of Medicine University of Illinois at Chicago Chicago, IL United States; ^6^ Department of Business Management Brooklyn College City University of New York New York, NY United States; ^7^ Health Policy Administration School of Public Health University of Illinois at Chicago Chicago, IL United States

**Keywords:** health policy, education, public health professional, education, medical, legislation, medical, problem-based learning, knowledge management, interdisciplinary communication

## Abstract

**Background:**

There is a dearth of advocacy training in graduate medical education in the United States. To address this void, the Legislative Education and Advocacy Development (LEAD) course was developed as an interprofessional experience, partnering a cohort of pediatrics residents, fourth-year medical students, and public health students to be trained in evidence-informed health policy making.

**Objective:**

The objective of our study was to evaluate the usefulness and acceptability of a service-based legislative advocacy course.

**Methods:**

We conducted a pilot study using a single-arm pre-post study design with 10 participants in the LEAD course. The course’s didactic portion taught learners how to define policy problems, research the background of the situation, brainstorm solutions, determine evaluation criteria, develop communication strategies, and formulate policy recommendations for state legislators. Learners worked in teams to create and present policy briefs addressing issues submitted by participating Illinois State legislators. We compared knowledge and attitudes of learners from pre- and postcourse surveys. We obtained qualitative feedback from legislators and pediatric residency directors.

**Results:**

Self-reported understanding of the health care system increased (mean score from 4 to 3.3, *P*=.01), with answers scored from 1=highly agree to 5=completely disagree. Mean knowledge-based scores improved (6.8/15 to 12.0/15 correct). Pediatric residency program directors and state legislators provided positive feedback about the LEAD course.

**Conclusions:**

Promising results were demonstrated for the LEAD approach to incorporate advocacy training into graduate medical education.

## Introduction

Since the 1970s, both US legislators and the public have shown diminished confidence in physician leadership [1,2]. In contrast, national health care and policy leaders are calling upon physicians to be trained in policy and advocacy in order to provide optimal care for their patients [3-5]. This shift in physician practice is emphasized by the American College of Graduate Medical Education. Milestones were implemented in 2015 as evaluation criteria for graduate medical education. For example, pediatric residents are expected to develop the ability to “advocate for quality patient care and optimal patient care systems” [6] and to “work in interprofessional teams to enhance patient safety and improve patient care quality” throughout their course of training [7].

There are very few published studies of curricula that train health care professionals in advocacy to provide optimal patient care [5,8,9]. Studies of these curricula conclude that involvement in an advocacy course increased the learner’s likelihood of pursuing future advocacy and that involvement of legislators led to more meaningful policy results [8,9]. However, we found no curricular descriptions of learners partaking in a dialogic process with legislators to understand values and issues and then using knowledge brokering to arrive at policy solutions and recommendations.

To address this void in health professional education, a multidisciplinary faculty committee at the University of Illinois at Chicago, USA, created the Legislative Education and Advocacy Development (LEAD) course to train pediatrics residents, public health students, and fourth-year medical students to think critically about health care, analyze policy, and communicate effectively about policy through the method of legislative briefing. The LEAD course sought to help learners to discern the actors and institutions involved in the policy-making process; critically examine the context of policy developments; appreciate how issues are placed on the policy-making agenda; understand the process of policy development, implementation, and modification; and apply dominant conceptual theories of the policy-making process to a critical health issue.

The LEAD curriculum therefore drew from previously established advocacy training programs to provide learners with the tools to understand and engage in health policy making [8,9]. The LEAD course incorporated project-based learning to enhance the learner experience and cultivate competencies outside of the traditional classroom setting [10,11]. Advanced organizers, which have been shown time and again to reduce cognitive load by providing methodological scaffolding, were an important addition to the course [12,13]. However, the LEAD course’s key innovation was the incorporation of knowledge brokering: bringing health science professionals, state legislators, and other stakeholders together to facilitate knowledge interaction and intermediation in the service-based learning process [14,15]. This approach went beyond the traditional linear knowledge-deliverance model because it was iterative and invoked active participation from all involved parties to develop new ideas and foster meaningful legislative action [16]. Our first aim with the LEAD curriculum was to measure learners’ demographics and changes in knowledge. We hypothesized that there would be significant improvement in our learners’ knowledge. Since there are very high correlations between symbolic political attitudes and political behaviors [17-19], our second aim was to measure learners’ attitudes before and after the course. We hypothesized that attitudes, which are symbolic in nature and thus resistant to change, would not shift significantly, but might change slightly [18-20]. Our final aim was to gather feedback from all invested parties: learners, pediatric residency program directors, and state legislators. We hypothesized that our program would be well received and considered a valuable addition.

## Methods

We used a single-arm pre-post study design to study the feasibility of the LEAD course and its impact on attitudes and knowledge among learners.

### Setting

We purposively invited pediatrics residents, fourth-year medical students, and public health students by email to participate in the 2-week LEAD course. A pediatrics faculty member with 3 years of policy experience and a public health faculty member with 20 years of policy experience were the instructors for the course. The course and study were conducted in February 2016. We expected learners to spend about 40 hours per week on their work. This time was divided thusly: 30 hours per week spent on modules and preparation with the group, and 10 hours per week on lectures and mock panels. Learners’ pre- and postcourse surveys were printed, self-administered, and anonymous to ensure privacy, and therefore completion of the surveys did not affect learners’ grades in the course. Additionally, only approved members of the research team had access to the surveys to ensure confidentiality.

We received ethical approval from the Institutional Review Board of the University of Illinois at Chicago (December 21, 2015, Research Protocol # 2015-1084). The study was consistent with the ethical standards of the Declaration of Helsinki. All learners provided verbal informed consent to participate in the study; this was obtained by the lead author and not recorded.

### Curriculum

The curriculum had two parts: didactics and experiential learning.

Learners participated in didactics, largely grounded in the works of Bardach, concerning background and landscape discovery, reiterative formulation of problem statements, and decision-making criteria [12]. Learners were instructed in the use of an advanced organizer that contained the core elements of a policy brief: issue statement, background, landscape, options and analysis, and final recommendation. Multimedia Appendix 1 shows this advanced organizer. The course objectives (Multimedia Appendix 2) were based on the advanced organizer. The curriculum focused on training learners to support recommendations with evidence and to use the advanced organizer for structure. Emphasis was also placed on developing legislator-derived, value-based criteria to evaluate each option and produce a final recommendation. An interactive overview of the state-level policy-formulation process was also provided. Learners participated in the policy-formulation process with extensive faculty mentorship and discussion. Beyond guidance on creating policy brief documents, participants also honed their oral presentation skills.

Concurrently, learners worked in 4 independent interdisciplinary teams to create briefs based on specific child health queries submitted directly from the state legislators. Learners discoursed both in live groups and virtually by cocreating briefs through Google Docs (Google Inc). Some examples of queries are lead abatement, gun control, access to home care services for disabled children, and licensure of professional midwives. In creating these briefs, learners used legislator values to create decision-making criteria, which guided research and policy analysis. Multimedia Appendix 3 shows an example of a decision-making chart. Learners presented their briefs during guided role play involving a panel of LEAD faculty and guest experts from the Department of Pediatrics and the School of Public Health, University of Illinois at Chicago. Additionally, participants identified and resolved common pitfalls encountered during the policy brief creation process [20]. The final product was a polished presentation with accompanying full-length and summarized policy briefs. Finally, learners formally presented their policy analysis and recommendations to state legislators and received feedback.

### Measures

#### Knowledge

We assessed knowledge with 15 questions on the pre- and postcourse surveys. These questions tested learners on factual data such as major US health care policies, components of a policy brief, and identification of state legislators and their governmental roles. Of these 15 questions, 13 were multiple choice questions with 4 to 12 answer choices, and 2 were free-response questions: “Who is your district’s state Senator?” and “Who is your district’s state Representative?” The highest possible correct total score was 15.

#### Attitudes

The pre- and postcourse surveys gave 13 attitude questions with possible answers ranging from 1=strongly agree to 5=strongly disagree. We analyzed each question separately. Content was adapted from 2 previously reported questionnaires on medical students’ and residents’ attitudes [21,22]. The 13 questions are tabulated below. Questions 1 and 2 were adapted from Stafford et al [22], questions 3 through 5, 7, 8, and 10 through 13 were from Emil et al [21], and questions 6 and 9 are original to this study.

#### Program Feedback

Learners were asked questions concerning quantity, quality, and engagement in past and present health policy instruction via the pre- and postcourse surveys. Questions 1 and 2 were rated from 1=excellent to 4=poor, questions 3 and 4 were rated from 1=excellent to 4=N/A (ie, not applicable), and questions 5 and 6 were rated from 1=strongly agree to 5=strongly disagree. To further measure feasibility and gauge interest among pediatric residency program directors, we presented the curriculum as a workshop at the Association of Pediatric Program Directors 2016 annual meeting and collected feedback. In addition to open-ended feedback, the 9 pediatric residency program directors who viewed the presentation were asked “Would you want this type of experience at your institution?” State legislators were queried in an open-ended format regarding their experience.

### Analysis

Due to an insufficient number of paired responses, we did not perform inferential statistical tests for the knowledge and attitudes questions. As applicable, we assessed program feedback data with a Wilcoxon signed rank test and otherwise assessed the feedback qualitatively for themes. We conducted statistical analyses using SAS version 9.4 (SAS Institute). All *P* values were 2-tailed. Thematic analysis of legislator and pediatric residency program director feedback was performed by 2 independent raters who evaluated all themes. Discrepancies were resolved by consensus.

## Results

A total of 10 learners provided pre- and postcourse surveys. We received 9 responses for demographic data (90% response rate), 5 precourse knowledge surveys (50% response rate), 7 postcourse knowledge surveys (70% response rate), 8 precourse attitude surveys (80% response rate), 7 postcourse attitude surveys (70%), and 10 sets of program feedback data (100% response rate). However, many of the pre- and postcourse attitude surveys were incompletely filled out by learners, and on further inspection it appeared this was partly due to secretarial issues, with some questions printed on the back of the page. We received qualitative feedback from 4 state legislators and 9 pediatric residency directors. Table 1 shows the demographics and characteristics of responders.

**Table 1:** Demographics and characteristics of participants in the Legislative Education and Advocacy Development course (n=10).

Characteristics		n	%
**Sex**			
	Male	6	60
	Female	2	20
	No response	2	20
**Age (years)**			
	22-25	1	10
	26-29	2	20
	30-33	2	20
	>34	3	30
	No response	2	20
**Race/ethnicity**			
	White (non-Hispanic)	1	10
	Black (non-Hispanic)	3	30
	Hispanic or Latino	2	20
	Asian or Pacific Islander	1	10
	Other	1	10
	No response	2	20
**Degrees earned**			
	MD/DO	5	50
	Bachelor’s degree	8	80
	Master’s degree	2	20
	PhD	0	0
	MPH	2	20
	No response	2	20

### Knowledge

**Figure 1:**
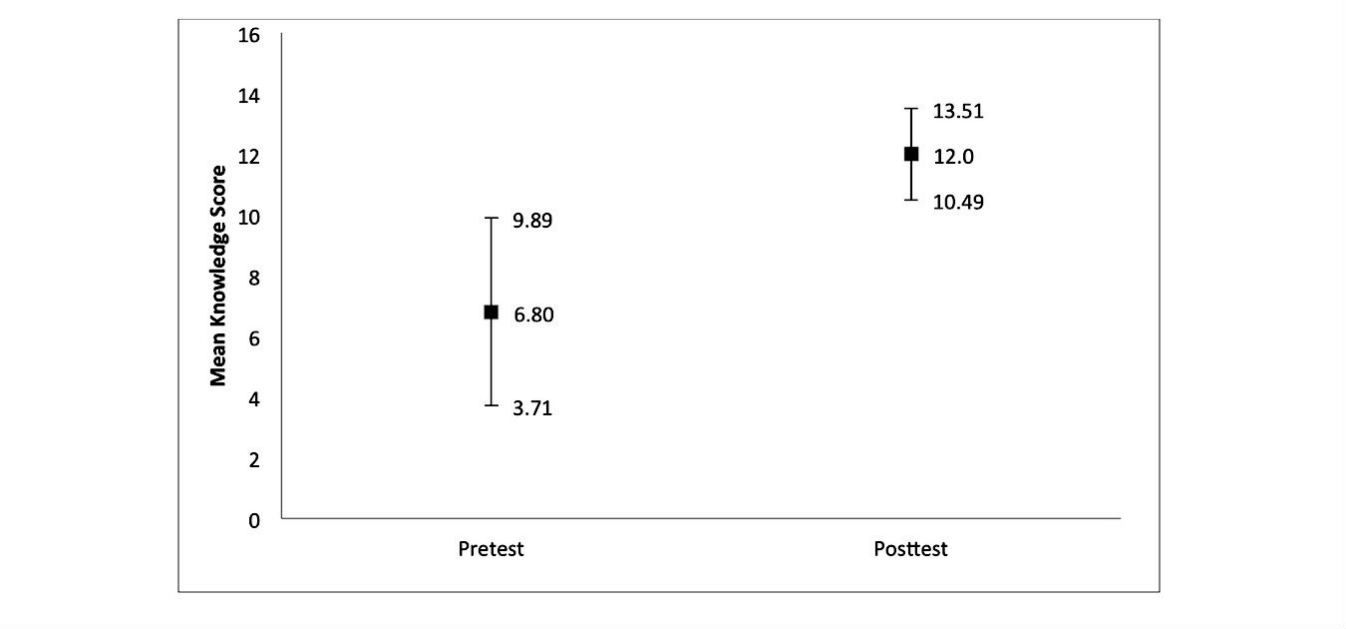
Mean and 95% CIs of pre- and postcourse knowledge scores.

Learners’ scores improved from a mean of 6.8 out of 15 to 12.0 out of 15 by the end of the course (Figure 1). Given the lack of overlap between the pre- and postcourse 95% confidence intervals, we noted a pattern toward improved knowledge. The lower limit of the postcourse knowledge score (10.49) did not include the upper limit of the precourse knowledge score (9.89). As there were only 3 sets of paired responses, we could not conduct an analysis with *P* values.

### Attitudes

Table 2 highlights the pre- and postcourse mean attitude scores. Attitudes were generally consistent from the pre- to postcourse surveys. Of the 13 items, 2 showed changes of 0.50 or more, toward greater recognition of the importance of health policy (question 6) and that the health care system should be government controlled rather than free market (question 9).

### Program Feedback

Table 3 shows the pre- and postcourse means for feedback measures of the LEAD course. Self-reported understanding of the health care system significantly improved, with mean Likert scores improved from 3.0 (fair) to 2.3 (good) (*P*=.01). Additionally, learners reported that health care policy instruction *prior* to the LEAD course was “little” in quantity and only “fair” in quality. Learners agreed they would be more likely to engage in health policy, and more likely to recommend to a colleague to engage in health policy learning, than they would have been 1 month prior to the end of the course.

**Table 2:** Mean pre- and postcourse attitude scores^a^, with number of responders.

Statement		Pretest mean (SD)	Posttest mean (SD)
1	It is part of my job as a physician to advocate for policy change on behalf of children.	1.38 (0.74) (n=8)	1.29 (0.49) (n=7)
2	I feel that my role as a health advocate extends beyond the individual patient(s) I am treating.	1.25 (0.46) (n=8)	1.14 (0.39) (n=7)
3	I plan to take leadership in health care policy issues as a physician.	1.75 (0.71) (n=8)	1.57 (0.54) (n=7)
4	I plan to support universal health care coverage as a physician.	1.50 (0.54) (n=8)	1.29 (0.49) (n=7)
5	I don’t expect to have any time to be active politically as a physician.	3.75 (0.89) (n=8)	3.86 (0.90) (n=7)
6	Health policy will have no effect on how I care for my patients.	4.50 (0.58) (n=4)	5.00 (0.00) (n=7)
7	The government should guarantee health care access for all citizens.	1.25 (0.50) (n=4)	1.29 (0.49) (n=7)
8	The government should provide health care access for all citizens, even if higher taxation is needed to generate sufficient revenue.	2.00 (1.41) (n=4)	1.57 (0.54) (n=7)
9	I would prefer my health care system to be “free market” rather than government controlled.	3.00 (0.00) (n=4)	3.57 (0.79) (n=7)
10	The government should regulate the prices of health care services.	2.50 (1.00) (n=4)	2.29 (0.49) (n=7)
11	Health care services would improve if the government had no involvement in health care.	4.25 (0.50) (n=4)	4.29 (0.95) (n=7)
12	All citizens should have access to the same standard of medical care without regard to their financial means.	1.50 (0.58) (n=4)	1.71 (1.50) (n=7)
13	All children should have access to the same standard of medical care without regard to their financial means.	1.00 (0.00) (n=4)	1.43 (1.13) (n=7)

^a^Answers ranged from 1=strongly agree to 5=strongly disagree.

**Table:** 

Questions	Precourse mean (SD)	Postcourse mean (SD)	*P* value
1. How would you rate your understanding of your health care system?	3.00 (0.67)	2.30 (0.48)	.01
2. How would you rate your understanding of health care systems in other “advanced/developed” countries?	3.30 (0.05)	3.00 (0.67)	.05
3. How would you rate the quantity of instruction on health care policy received in your current training program?	2.70 (0.67)	N/A^b^	N/A
4. How would you rate the quality of instruction on health care policy received in your current training program?	2.89 (1.27)	N/A	N/A
5. The current likelihood of my engaging in health policy activity/activities has increased compared to 1 month ago.	N/A	2.29 (0.82) (n=7)	N/A
6. The current likelihood of my recommending that my colleagues engage in health policy learning has increased compared to 1 month ago.	N/A	2.43 (1.41) n=7	N/A

^a^Questions 1 and 2 were rated from 1=excellent to 4=poor, questions 3 and 4 were rated from 1=excellent to 4=N/A, and questions 5 and 6 were rated from 1=strongly agree to 5=strongly disagree.

^b^N/A: not applicable.

We queried 4 state legislators about their experience with the LEAD course, and their responses were positive. Specifically, 2 state legislators expressed themes of efficacy. For example, 1 legislator wrote that this was “a thoughtful and well-researched brief that greatly improved my understanding in the area.” All state legislators expressed a desire to continue participating. For example, 1 legislator wrote, “I look forward to working with the learners again next year.” Of 9 pediatric residency program directors queried at the national conference, 8 (89%) said yes and 1 (11%) said maybe, regarding their desire for this type of course at their institution. Qualitatively, they found the experience insightful, were interested in viewing the didactics, and would like to incorporate LEAD into their training program.

## Discussion

### Principal Findings

The hypotheses for our LEAD course pilot study were all supported. We found that knowledge improved from pre- to postprogram. We found that attitudes were generally consistent from pre- to postprogram. We found that the pilot was well received by learners who took the course, pediatric residency program directors who may choose to implement the course, and the state legislators who participated in the course.

Knowledge improved meaningfully when learners’ scores improved (6.8/15 to 12.0/15) on the postsurvey questionnaire. Knowledge outcomes among health policy training programs for medical students and residents to date have been self-assessed [5,9]. One study using learners’ self-assessed knowledge improvement found a statistically significant increase in 5 areas of knowledge [9]. A previous study asked learners to self-assess their knowledge before and after course completion across several topics, including quality of and access to care, Medicaid and Medicare, and the role of government in health policy [9]. Both methods of evaluation have shown improvement in knowledge. Our findings within the LEAD program were consistent with other approaches demonstrating improved knowledge.

As hypothesized, scores on attitude questions for LEAD learners did not generally change between pre- and postcourse. This was likely a product of the already extreme Likert responses at baseline and the small sample, which self-selected into a policy course. Categorically stable attitudes that are held over time tend to better predict behavior than attitudes that change [23]. Two questions demonstrated variation: question 6 (greater recognition of the importance of health policy) and question 9 (health care system should be government controlled rather than free market), which both moved 0.50 points along the scale. While the cohort of learners who partook in the LEAD course generally displayed categorically stable attitudes, one potential caveat to this stability and general trend toward “progressive” attitudes was an apparent internal inconsistency between learners’ signaled general support for universal health care and their support for financial means testing. Scores for question 12 showed that learners were more likely to consider “financial means testing” after the course. Although this may reflect a dichotomy between principles and the means of achieving principled goals, policy “targeting” (eg, financial means testing) is not necessarily juxtaposed to universalism [24]. It is possible that these learners were signaling greater nuance in their understanding of redistributive policy, a product of engaging with contradictory forces in a highly complex system [25].

Measuring attitudes is important, since attitudes may correlate closely with long-term behavioral outcomes in general [17-19], and specifically are thought to be indicators of health professional behaviors [26]. A previous study gauged the attitudes of learners in California, USA and Ontario, Canada [23]. The LEAD cohort of learners more closely reflected the participants of the Ontario than of the California cohort, but the LEAD learners were more agreeable than both the Ontario and California cohorts. For instance, both Ontario and California learners “agreed” while LEAD learners “strongly agreed” that they planned to become involved and take leadership in health care policy issues as a physician. We suggest that demography and self-selection into a policy course are possible reasons for attitudes discrepant with those previously reported [23].

Another focus of our study was to measure feedback from the 3 key types of players in the LEAD course: learners who took the course, pediatric residency program directors who may choose to implement the course, and the state legislators who participated in the course. There was broadly positive feedback from learners, pediatric residency program directors, and state legislators. More specifically, evaluative data from learners suggested that their understanding of the health care system improved, and prior to our course, their health care policy training was quite limited. In addition, the learners indicated a “good” likelihood of both engaging in health policy activities and recommending that a colleague engage in health policy learning. This is important because we know that learners’ subjective opinions about a course directly translate to both their long-term behavioral changes and their underlying satisfaction with their education [27]. These feedback data from pediatric residency program directors is a measure of external validation, as these program directors were from different academic centers and therefore may have provided greater objectivity concerning programmatic strengths [28]. As demonstrated by responses from the pediatric residency program directors, LEAD would be a desirable addition to other pediatric residency programs. The LEAD curriculum can be easily exported to diverse residency programs, as it has no specific geographical or institutional requirements.

Although legislators have previously signaled a desire to work with undergraduates, none have been surveyed in the context of graduate medical education [29]. As demonstrated by the positive response of the state legislators, it is reasonable to assume that we brokered a meaningful interaction between the learners and legislators. Therefore, it is likely that state legislators who are interested in improving the health of their communities would be willing to participate with future iterations of LEAD. To further institutionalize this approach, educational leaders can work with legislative leaders and their staff to strengthen the didactic and formative learning approach. For instance, in Illinois, the LEAD leadership team worked closely with the leaders of the legislative caucuses—House and Senate Democrats and Republicans—to identify issues and active bills that might serve as centerpieces for engaged interprofessional service-based learning.

### Limitations

This study has several limitations. First, the sample size was small. Second, we did not use a control group. This limited the ability to assess attitudes and knowledge of those medical students, residents, or public health students not participating in an intensive advocacy experience. Third, the lack of responses limited the ability to perform certain inferential statistical tests. Fourth, we did not collect data on how learners used their time; this would have been valuable to examine and perhaps compare with other advocacy training programs. Future research should study this topic with a larger sample and a control group.

### Conclusion

There were promising results from the LEAD course as an acceptable and useful tool incorporating advocacy training into graduate medical education in the United States. The LEAD curriculum should be considered by institutions and programs seeking to help generate a new cadre of policy leaders from within the health professions.

## Appendix

Multimedia Appendix 1 Advanced organizer.

Multimedia Appendix 2 Course objectives.

Multimedia Appendix 3 Decision-making chart.

## Abbreviations

LEAD: Legislative Education and Advocacy Development
