# Supplementary material for: Systems-Based Training in Graduate Medical Education for Service Learning in the State Legislature in the United States: Pilot Study
Source: JMIR Med Educ. 2017 Oct 17;3(2):e18. doi: 10.2196/mededu.7730 (PMC5663953; doi:10.2196/mededu.7730)
Supplement: Multimedia Appendix 1 [file mededu_v3i2e18_app1.pdf]

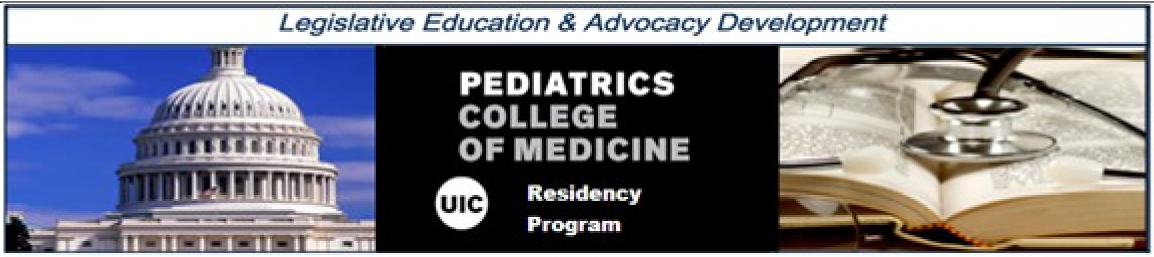

1. **ISSUE STATEMENT:**

2. BACKGROUND:

3. LANDSCAPE:

4. OPTIONS:

5. OPTIONS ANALYSIS:

6. RECOMMENDATION/IMPLICATIONS:
