# Supplementary material for: Systems-Based Training in Graduate Medical Education for Service Learning in the State Legislature in the United States: Pilot Study
Source: JMIR Med Educ. 2017 Oct 17;3(2):e18. doi: 10.2196/mededu.7730 (PMC5663953; doi:10.2196/mededu.7730)
Supplement: Multimedia Appendix 2 [file mededu_v3i2e18_app2.pdf]

Read and understand legislative language.

Incorporate political factors into analysis.

Work under time constraints and to allocate analytic resources.

Obtain policy-relevant data, through efficient search techniques and persistence.

Cope with uncertainty in a policy context by being flexible, tolerating false starts and dead ends.

Advocate for uncomfortable positions.

Learn to identify limitations of analysis and work within constraints.

Collect, analyze, and assimilate information in an iterative and interactive knowledge building process
