# Supplementary material for: Systems-Based Training in Graduate Medical Education for Service Learning in the State Legislature in the United States: Pilot Study
Source: JMIR Med Educ. 2017 Oct 17;3(2):e18. doi: 10.2196/mededu.7730 (PMC5663953; doi:10.2196/mededu.7730)
Supplement: Multimedia Appendix 3 [file mededu_v3i2e18_app3.pdf]

VALUES (decision-making criteria are derived from values and weighted by importance):

1.

2.

3.

4.

5.

	Option #1	Option #2	Option #3	Option #4	Option #5
Decision Criteria #1					
Decision Criteria #2					
Decision Criteria #3					
Decision Criteria #4					
Decision Criteria #5					
Total:					
